# Supplementary material for: Novel Driver Strength Index highlights important cancer genes in TCGA PanCanAtlas patients
Source: PeerJ. 2022 Aug 11;10:e13860. doi: 10.7717/peerj.13860 (PMC9375969; doi:10.7717/peerj.13860)
Supplement: Supplemental Information 2 [file peerj-10-13860-s002.zip › COHORTS/genes plots/cohorts/2021_8_16_14_9_distribution_cohorts_LUSC.pdf]

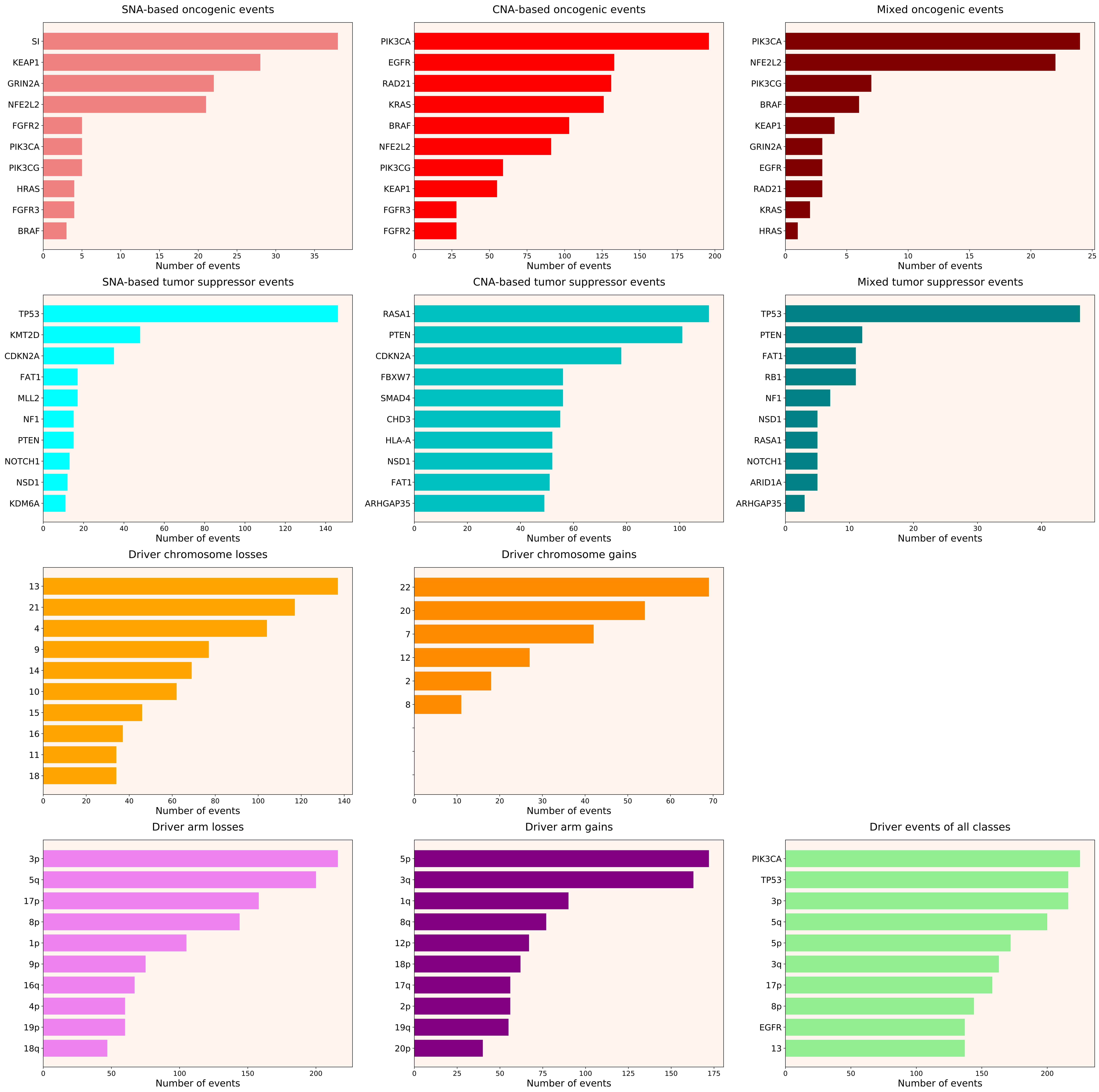

SNA-based oncogenic events
SI
KEAP1
GRIN2A
NFE2L2
FGFR2
PIK3CA
PIK3CG
HRAS
FGFR3
BRAF
0 5 10 15 20 25 30 35
Number of events
CNA-based oncogenic events
PIK3CA
EGFR
RAD21
KRAS
BRAF
NFE2L2
PIK3CG
KEAP1
FGFR3
FGFR2
0 25 50 75 100 125 150 175 200
Number of events
Mixed oncogenic events
PIK3CA
NFE2L2
PIK3CG
BRAF
KEAP1
GRIN2A
EGFR
RAD21
KRAS
HRAS
0 5 10 15 20 25
Number of events
SNA-based tumor suppressor events
TP53
KMT2D
CDKN2A
FAT1
MLL2
NF1
PTEN
NOTCH1
NSD1
KDM6A
0 20 40 60 80 100 120 140
Number of events
CNA-based tumor suppressor events
RASA1
PTEN
CDKN2A
FBXW7
SMAD4
CHD3
HLA-A
NSD1
FAT1
ARHGAP35
0 20 40 60 80 100
Number of events
Mixed tumor suppressor events
TP53
PTEN
FAT1
RB1
NF1
NSD1
RASA1
NOTCH1
ARID1A
ARHGAP35
0 10 20 30 40
Number of events
Driver chromosome losses
13
21
4
9
14
10
15
16
11
18
0 20 40 60 80 100 120 140
Number of events
Driver chromosome gains
22
20
7
12
2
8
0 10 20 30 40 50 60 70
Number of events
Driver arm losses
3p
5q
17p
8p
1p
9p
16q
4p
19p
18q
0 50 100 150 200
Number of events
Driver arm gains
5p
3q
1q
8q
12p
18p
17q
2p
19q
20p
0 25 50 75 100 125 150 175
Number of events
Driver events of all classes
PIK3CA
TP53
3p
5q
5p
3q
17p
8p
EGFR
13
0 50 100 150 200
Number of events
